# Relationship between Biological Maturation, Physical Fitness, and Kinanthropometric Variables of Young Athletes: A Systematic Review and Meta-Analysis

**DOI:** 10.3390/ijerph18010328

**Published:** 2021-01-05

**Authors:** Mario Albaladejo-Saura, Raquel Vaquero-Cristóbal, Noelia González-Gálvez, Francisco Esparza-Ros

**Affiliations:** 1 Kinanthropometry International Chair, Catholic University San Antonio of Murcia (UCAM), Av. de los Jerónimos 135, 30107 Murcia, Spain; mdalbaladejosaura@ucam.edu (M.A.-S.); fesparza@ucam.edu (F.E.-R.); 2 Faculty of Sport, Catholic University San Antonio of Murcia (UCAM), Av. de los Jerónimos 135, 30107 Murcia, Spain

**Keywords:** growth, maturation, kinanthropometry, youth sports, performance

## Abstract

There is a growing interest in knowing the relationship between biological maturation and sport performance-related variables of young athletes. The objective of this study is to analyze the relationship between biological maturation, physical fitness, and kinanthropometric variables of athletes during their growing period, according to their sex. The systematic review and meta-analysis followed the Preferred Reporting Items for Systematic Review and Meta-Analysis (PRISMA) statement and the search protocol was registered in PROSPERO, code: CRD42020208397. A search through the PubMed, Web of Sciences, and EBSCO databases was performed. A total of 423 studies were screened and 13 were included in the meta-analysis. The meta-analysis was completed by using the mean and standard deviation of each variable according to each maturation status (early, on time, or late). Differences depending on maturation were found on physical fitness, with better results in the advanced maturational groups in the male population (standard mean difference (SMD) = 0.17–2.31; *p* < 0.001–0.05). Differences depending on maturation were found for kinanthropometric variables in males (SMD = 0.37–2.31; *p* < 0.001–0.002) and height and body mass in females (SMD = 0.96–1.19; *p* < 0.001). In conclusion, the early maturation group showed higher values in kinanthropometric variables and better results in physical fitness, highlighting the importance of the maturational process in the talent selection programs. Despite that, more research is needed to clarify the relationship of maturation with the other variables on female populations and the changes in the muscle and bone variables during the maturation processes of both sexes.

## 1. Introduction

The early identification of young talents brings certain benefits to the clubs that implement this process. Among the advantages can be found an early specialization in the skills and capacities of the sport, the incorporation of young players to the high-level team, or long-term economic security [1,2,3]. In addition, in sports with smaller incomes, the early identification and monitoring of sports talents are of vital importance for the optimization of economic resources [3]. As a consequence, there has been a growing interest in creating models that allow for the identification and even prediction of future talents among young athletes in the last decade [2,3].

Talent in sports could be defined as the ability to provide a correct answer to the specific demands of sports performance [1]. Among the factors analyzed in the talent identification programs in sports, physical performance plays an important role, as it is considered one of the most determinant factors in the future sports performance of young athletes [4]. Other main components of talent identification models are the kinanthropometric variables, due to their relationship with sports performance [5]. In fact, kinanthropometry has been used to characterize the morphological requirements of different sports disciplines [6,7,8]. However, it must be taken into account that the reference values observed in adult elite athletes should not be extrapolated to athletes who are in the process of growing since the maturation stage can affect kinanthropometric characteristics and physical performance [9].

Maturation, in relation to human growth, refers to the time required and the process of change until the adult maturation state is reached [10]. The physical and physiological changes that occur during the progress of biological maturation evolve at a different pace, depending on the subject [10]. Due to the influence of biological maturation in sports performance, it seems necessary to evaluate the maturation stage in which the adolescent athletes are currently in, with the peak height velocity (PHV) being one of the most useful indicators of the maturation stage [10,11]. Furthermore, there are differences between males and females in terms of the age at which PHV begins and the age at which the maximum growth peak occurs (age at peak height velocity, APHV). It is common to observe this occurring between 9.3 and 15 years of age in females and between 12 and 15.8 years of age in males [10].

The gold standard for estimating PHV is the radiological evaluation of specific bones in the hand and wrist [12,13]. This method is an accurate way to assess the maturation of the adolescents and is widely used and validated [12,13], but it requires very experienced researchers to ensure the validity and reliability of the results, and the equipment needed is inaccessible in most of the cases [14]. Furthermore, other alternative methods have been used, such as kinanthropometric variables through regression formulas [11], due to its validity and reliability, with other advantages such as it is easy, inexpensive, transportable, and innocuous [15,16]; or the evaluation of maturation based on the development of secondary sexual characteristics [17,18]. This latter method relies on a self-reported assessment based on secondary sexual factors influenced by maturation, and it is easy to do by the subjects [17,18]. However, it is a subjective evaluation [19], and in determining situations it could be considered personally intrusive [11].

There is a growing interest in knowing the relationships between biological maturation, kinanthropometric variables, and the physical performance of young athletes due to its influence on these parameters, which are related to sports performance [4]. In the academic training stages, it has been observed that young athletes who mature earlier than their peers of the same chronological age are more likely to be selected for high-performance programs [20], although they do acquire a high-level performance when the growing period ends [21]. This is because during the maturation process physical and physiological changes occur that affect sports performance, offering to mature earlier a competitive advantage in most cases [10]. Along this line, and regarding muscle and bone tissue, there is a significant increase due to the hormonal changes that are typical of adolescence, and this is more marked when there is a systematic practice of physical exercise [10,22]. However, this increase does not occur equally in both sexes, with similarities observed in the early stages of development and a greater increase in strength and muscle mass in males at the peak of growth and late stages of development [23,24]. As for fat mass, which has been one of the most-analyzed variables due to its relation with sports performance [25,26,27,28], it has been observed that a greater accumulation of fat is related to earlier maturation in females and later in males [29,30].

Regarding physical conditions, it has been observed that young athletes who mature earlier have better results in endurance tests [31], the strength of both upper and lower limbs [9], and sprint ability [32], with these being decisive characteristics for sports performance. A similar tendency has been found in both sexes [33,34]. The better results shown by the early maturers are determinant factors in future sports performance, as the speed and power production abilities have been demonstrated to be a discriminatory factor between elite and non-elite athletes [35]. These physical condition variables improve during puberty, which enhances the selection of early maturers for talent identification programs when biological maturation is not assessed [36]. Despite that, some variables, like endurance, improve the most after PHV [37].

However, despite the clear influence that the differences in maturation may have on the physical conditions and kinanthropometric characteristics of young athletes, there is some discrepancy on the specific weight of these variables for explaining the differences in performance depending on maturation [38,39,40,41]. Furthermore, a great heterogeneity has been found between studies in terms of participants, sports analyzed, and tests included [9,32,33,34,42]. Therefore, the objective of this study is to analyze the relationship of biological maturation, physical fitness, and kinanthropometric variables of athletes in the growing period, according to their sex.

## 2. Materials and Methods

This systematic review and meta-analysis followed the Preferred Reporting Items for Systematic Review and Meta-Analysis (PRISMA) statement [43], and the search strategy, inclusion criteria, and additional information were registered in advance with the international prospective registry of systematic review PROSPERO (code: CRD42020208397). 

### 2.1. Search Strategy

A search through the PubMed, Web of Sciences, and EBSCO databases was performed up to 18 September, 2020. The keywords used were “biological matur*”, “sport performance”, “training”, “anthropometry”, “kinanthropometry”, “body composition” and “somatotype”, combined with the linkers “AND” and “OR”: (Biological matur* AND (sport performance OR physical fitness) AND (anthropometry OR kinanthropometry OR body composition OR somatotype). Studies that examined the relationship of biological maturation with different types of physical performance or kinanthropometric variables were included for the meta-analysis.

The inclusion criteria were (a) observational studies; (b) outcome measurements based on physical performance, kinanthropometric variables, or both; (c) results divided by maturity group; (d) participants aged 9 to 15 years old for females and 12 to 16 years old for males, as the age range when APHV occurs [10]; (e) written in English or Spanish; (f) active population participating in a specified sports discipline. The exclusion criterion was groups with less than five participants [44]. 

### 2.2. Data Extraction and Risk of Bias

Two reviewers (M.A.-S. and R.V.-C) performed the search independently, screened the titles and abstracts from the search results, and reviewed the full text selected before the inclusion in the meta-analysis. A third reviewer (F.E.-R.) was consulted to resolve any disagreement regarding inclusion. To determine the inter-rater reliability of the reviewers, Cohen’s Kappa [45] was calculated, showing a strong level of agreement (Kappa = 0.901).

### 2.3. Quality Assessment and Risk of Bias

The Strengthening Reporting of Observational Studies in Epidemiology (STROBE) statement [46] was used to assess the quality of the studies included. Quality assessment was performed by two reviewers (M.A.-S. and R.V.-C). A third reviewer was consulted to resolve any disagreements (F.E.-R.). Egger’s [47] bias statistics and Rosenthal’s [48] fail-safe N was used to assess the risk of bias and funnel plots were created (Figure S1). When a meta-analysis is based on a small number of studies, the capacity of Egger’s test to detect bias is limited [49]. Therefore, this test must be performed when there are at least ten studies included in the meta-analysis [47].

### 2.4. Statistical Analysis

The statistical analysis and meta-analysis were performed using the Comprehensive Meta-Analysis program (version 3, Englewook, Bergen County, NJ, USA). The meta-analysis was completed for continuous data by using the mean and standard deviation of each variable and according to each maturation status (early, on time, or late). This information was directly extracted from the studies. The analysis was performed when at least two groups were included for the same variable. When a study included more than one group separated by age range or sport, all groups were included in the analyses. For studies that did not include the necessary data, the standard desviation (SD) was calculated and imputed when possible using standard errors and confidence intervals. The DerSimonian-Laird (Cohen) pooling method was used, and heterogeneity was assessed using the Cochrane Q test (Chi2), Higgins I2, and significance (*p*) to determine the appropriateness of the application of a fixed or random-effect model for the pooled analysis [50]. A meta-analysis with a random-effects model was performed to infer the pooled estimated standardized mean difference (SMD) [51,52]. DerSimonian-Laird (Cohen) was interpreted using Cohen’s [53] as small (0 to 0.2), medium (0.3 to 0.7), and large (>0.8). The significant differences were determined at a level of *p* < 0.05.

## 3. Results

### 3.1. Data Search and Characteristics of the Studies

**Figure 1:**
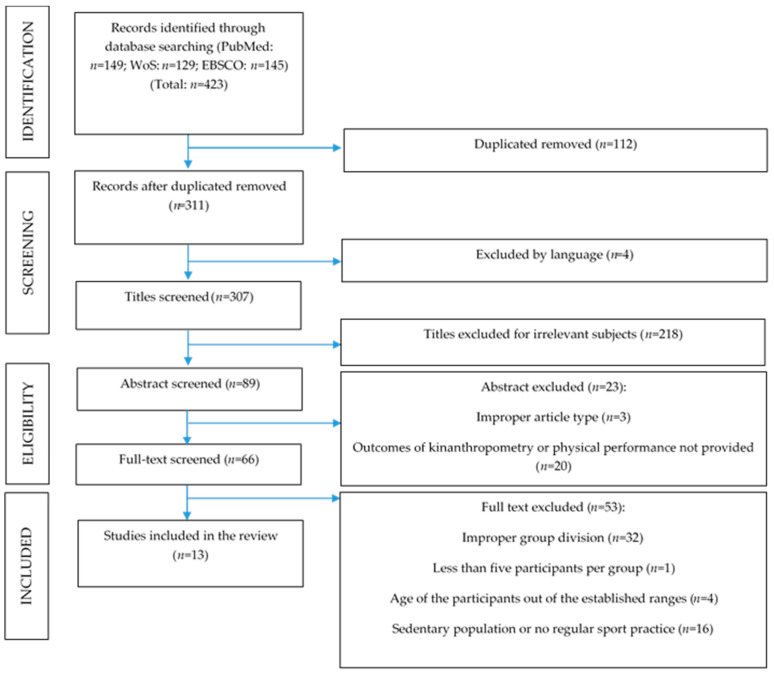
Flow diagram for the search and screened and included articles.

A total of 423 studies were screened and 13 were finally included in the meta-analysis (Figure 1). 

The characteristics of the analyzed studies can be observed in Table 1. The quality of the selected studies, assessed with the STROBE scale, can be observed in Table 2. All the studies followed a descriptive design (STROBE scale range 15–20), involving a total of 1431 subjects (1323 males; 108 females). The mean sample size was 79.50 ± 43.13 (range 28–168). Two studies were carried out with females (15.38%) [34,54], and 11 were carried out with males (84.62%) [9,31,32,33,42,55,56,57,58,59,60]. The most represented sport was football (*n* = 5; 38.46%) [32,55,57,59,60], followed by basketball (*n* = 3; 23.07%) [31,33,54] and handball (*n* = 2; 15.38%) [42,58]. 

Six studies (46.15%) used the APHV estimation formula based on kinanthropometric measurements [9,31,33,42,56,58], five studies (38.46%) used X-ray radiographic methods [32,34,55,57,60], and two studies (15.38%) used sexual maturity methods [54,59] to assess the maturity of the sample. The majority of the studies divided the sample into three maturational groups (*n* = 10; 76.93%) [9,31,32,33,42,54,55,57,59,60], and three of them into more mature or less mature groups (*n* = 3; 23.07%) [34,56,58].

### 3.2. Physical Fitness Results

Up to 26 different tests were used in the 13 articles included in the analysis. All the studies included in the meta-analysis of the relationship between maturation and physical fitness tests were conducted with males (*n* = 11; 84.62%). None of the physical fitness tests were repeated in the two articles including females (*n* = 2; 15.38%) and the meta-analysis could not be performed in this population group.

Table 3 shows the mean and standard deviation of each physical fitness variable according to early, on time, and late maturation, and meta-analysis data (SMD: standardized mean difference; 95%CI: 95% confidence interval, a: test for overall effect, p: significance). For the early vs on time analysis, and the for early vs late analysis, a positive SMD indicates a higher value for early maturation than on time or late maturation. For the on-time vs late analysis, a positive SMD indicates a higher value for the on-time maturation group than the late maturation group. The meta-analysis showed statistical differences between the early and on-time maturation groups in the squat jump test (SJ) (SMD = 0.23; *p* = 0.04), countermovement jump (CMJ) (SMD = 0.17; *p* = 0.04), medicine ball throw (SMD = 0.99; *p* < 0.001), and handgrip strength (SMD = 1.31; *p* < 0.001), with a better performance for the early maturers group. The analysis of the differences between the early and late maturation groups showed statistical differences in the CMJ (SMD = 0.38; *p* = 0.03), medicine ball throw (SMD = 1.58; *p* < 0.001), handgrip strength (SMD = 2.31; *p* < 0.001), sprint (SMD = −0.94; *p* < 0.001), and agility *t*-test (SMD = −0.52; *p* = 0.001), with the early maturers group obtaining better results. The sprint test (SMD = −0.42; *p* = 0.05), handgrip strength (SMD = 1.09; *p* < 0.001), and medicine ball throw (SMD = 0.89; *p* < 0.001) tests showed statistical differences when the on-time and late groups were compared, with the on-time maturer group showing better results. The Yo-Yo test and the sit and reach test did not show statistical differences in any of the groups compared. 

**Figure 2:**
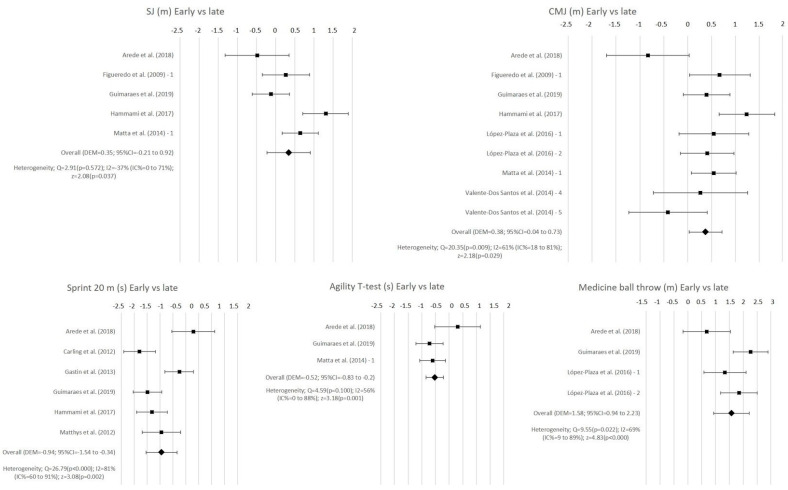
Forest plot of early and late maturation for physical fitness tests.

Forest plots were created in the cases when there were at least three studies and when at least one of the comparisons between the variables was significant (early vs. late, early vs. on time, or on time vs. late). Figure 2 shows forest plots for SJ, CMJ, medicine ball throw, sprint 20 m, and agility *t*-test. Egger’s test did no evidence publication bias by CMJ on time vs. late (SE = 0.514; 95%CI = −0.197–1.296; *p* = 0.128), although light evidence of publication bias by CMJ early vs on time was reported (SE = 0.643; 95%CI = 0.031–2.180; *p* = 0.045).

The most utilized test was the countermovement jump (CMJ), found in 11 articles (84.61%). Two articles (18.18%) found statistical differences for this variable between early and on-time maturation groups [9,55] (Table S1); four articles (36.36%) found differences between early and late maturation groups [33,55,58,59] (Table S1); and only one (9.09%) between on-time and late maturation groups [55] (Table S1). The squat jump test (SJ) and sprint test for different distances were used in seven articles (53.85%, respectively). In the SJ test, statistical differences were found between early and on time groups in one article (14.28%) [55] (Table S2), and between early and late matures in two of them (28.57%) [58,59] (Table S2). None of the articles analyzed found differences in the on-time and late groups in the SJ test (0%) (Table S2). For the sprint test, differences were found in two articles (28.57%) when early and on time maturation groups were compared [31,32] (Table S3); in four articles (57.14%) when early and late maturation groups were compared [31,32,42,58] (Table S3), and in two (28.57%) articles when the groups compared were on time versus early [32,42] (Table S3). For the Yo-Yo intermittent recovery test (*n* = 6; 46.15%), two articles (33.33%) found statistical differences between early and on-time groups [31,60] (Table S4), four articles (66.67%) found differences when the early and late maturation groups were compared [31,33,55,60] (Table S4), and two articles (33.33%) found differences between on-time and late groups [55,60] (Table S4). The handgrip strength test (*n* = 4; 30.76%) showed statistical differences between all three groups in all the articles (100%) that analyzed this variable [31,34,42,57] (Table S5). All the articles that included the medicine ball throw (*n* = 3; 23.08%) in the physical fitness test found statistical differences between groups (66.67%), except one (33.33%) [33] (Table S6). In the agility t-test (*n* = 3; 23.08%), statistical differences were found for the early and on-time groups in one article (33.33%) [31] (Table S7), for the late and early groups in two articles (66.67%) [31,59] (Table S7), and for the on-time and late groups in one article (33.33%) [59] (Table S7). No differences were found in the sit and reach (SR) test (*n* = 2; 15.38%) except for one article in the comparison between early and late matures (50%) [9] (Table S8). More detailed information about the differences between groups in the physical fitness test, including the sample sizes of each group in the different studies and the specific weight (%), can be found in the supplementary materials (Tables S1–S8).

### 3.3. Kinanthropometric Variables Results

Throughout the 13 articles analyzed, a total of 11 kinanthropometric variables were used. Table 3 shows the results of the differences between groups for the kinanthropometric variables, including the means and standard deviations, standardized mean difference (SMD), 95% CI, overall size effect (Z), and significance (*p*). The meta-analysis was performed in five kinanthropometric variables in males (body mass, height, sitting height, body mass index (BMI), fat mass percentage) due to the lack of information provided about the other variables. From these, only body mass, height, and BMI could be included in the analysis of the articles performed with females, and there was no possibility for including the on-time maturation group.

In males, all the variables used to compare the three groups showed statistical differences (SMD = 0.37–2.56; *p* < 0.001–0.02), showing that early maturation is related to higher values of body mass, height, sitting height, BMI, and fat mass percentage. In females, there were statistical differences between early and late maturers in body mass (SMD = 0.96; *p* < 0.001) and height (SMD = 1.19; *p* < 0.001), finding higher values in these variables for the more mature individuals (Table 3).

**Figure 3:**
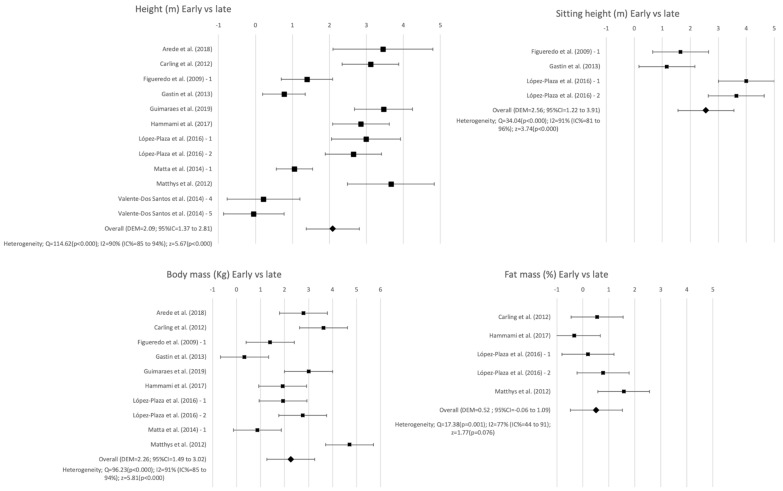
Forest plot of early and late maturation for kinanthropometric variables.

Figure 3 shows forest plots for height, sitting height, body mass, and fat mass. Just as with the fitness variables, the forest plots were created when there were at least three studies and when at least one of the comparisons between the variables were significant (early vs late, early vs on time, or on time vs late). No evidence of publication bias was reported by Egger’s test for height early vs on-time (SE = 0.152; 95%CI = −1.876–2.925; *p* = 0.637), height early vs late (SE = −0.083; 95%CI: −3.183–2.512; *p* = 0.798), height on time vs. late (SE = 0.540; 95%CI = −0.137–2.883; *p* = 0.070), or weight early vs. late (SE = −0.489; 95%CI = −3.421–0.632; *p* = 0.151).

All of the articles provided information about the height and body mass of the participants (*n* = 13; 100%). Statistical differences were found between the three groups for the height except in one article (7.7%) [60] (Tables S9 and S14). All the articles reported statistical differences in body mass between groups except Matta et al. (7.7%) [59] when the early and on time groups were compared, and Gastin et al. (7.7%) [56] when early and late groups were compared (Tables S10 and S15). The fat mass percentage was assessed in seven articles (53.84%). Only one article found statistical differences in fat mass percentage between early and on time groups (14.28%) [42] (Table S11), three articles found statistical differences between early and late groups (42.86%) [9,32,42] (Table S11), and four articles found differences between on-time and late groups (57.14%) [9,32,42,57] (Table S11). Sitting height was used in six articles (46.16%), but only three included the data in the results [9,55,56]. Statistical differences were found for all the groups in the sitting height (100%) [9,55,56] (Table S12). The BMI differences were analyzed in three articles (23.07%). Statistical differences were found for all the groups, except in the comparison between late and early maturers according to Sogut et al. (33.33%) [34], and in the comparison between on-time and late maturers according to López-Plaza et al. (33.33%) [9] (Tables S13 and S16). More detailed information about the differences between groups for the kinanthropometric variables, including the sample sizes of each group in the different studies and the specific weight (%), can be found in the supplementary materials (Tables S9–S16). 

## 4. Discussion

The main objective of the present review with meta-analysis was to show the relationships between different biological maturation stages and the physical fitness of young athletes. The biological maturation showed to have a statistically significant relationship with physical fitness in males. When a comparison between different maturation groups was performed, a tendency to obtain better results was observed when the maturation process was more advanced. Analyzing the overall differences between maturation groups, significant differences were found in medicine ball throw and handgrip strength tests. Furthermore, significant differences were found in CMJ between early and on time, and early and late groups; in sprint between early and late, and on time and late groups; in the SJ test between early and on time groups; and agility *t*-tests between early and late groups. All the tests where the differences were found were related to the ability to produce power and strength [9,33]. The production of strength is dependent on neural factors in the early stages of the training adaptations, but it is also highly influenced by an increase in muscle mass [62,63]. Among the factors that positively affect the production of muscle power, it has been observed that one of the key factors is muscle mass, with a relationship existing between the increase of muscle mass and the production of power [64,65]. Testosterone, which is an index of the hypothalamic–pituitary–gonadal axis, a primary neuroendocrine system involved in advancing puberty, has a marked increase during adolescence in males [65]. After adolescence, male subjects could have up to 30 times more testosterone [66]. This steroid hormone plays a determinant role in sports performance, because of the effect that it produces in lean muscle mass gain [66,67]. This could explain the better results obtained by the more matured subjects with respect to their less matured peers in the physical fitness test that directly depended on the muscle mass. However, from the analyzed studies, only two studies included variables related to muscle mass [9,60]. López-Plaza et al. [9] analyzed the percentage of muscle mass, without finding differences between groups. Due to the use of a relative value to assess the muscle mass (%) instead of the absolute weight of muscle mass (kg) [9], and together with the fact that other body masses such as fat mass can increase during the period of growth, as found in the present study, the use of muscle percentages could indicate that some information is missing about the absolute differences between maturation groups as related to muscle mass, which could explain the differences in the performance. Valente dos Santos et al. [60] assessed the fat-free mass by subtracting the fat mass from the total body mass. The fat-free mass includes various tissues such as bone mass, skin mass, or visceral mass, apart from muscle mass [7,68], which can lead to an underestimation of the changes produced in muscles. Future research studies could clarify the relationship between muscle mass and performance in young athletes.

No differences were found in the Yo-Yo test or the sit and reach test. The Yo-Yo test is used to assess the VO_2_ Max, as the main variable of aerobic capacity [33,55]. The aerobic performance seems to be more influenced by training variables than by other variables [69,70,71], and the age at the peak of better performance has been shown to be far from adolescence development [72], which could be an explanation for the lack of differences between maturation groups. The sit and reach test assesses the extensibility of the hamstring muscles [73]. This musculature tends to shorten due to the histological and biomechanical factors but can also be influenced by the age and the practice of certain sports [74,75]. In contrast, extensibility seems to be sensitive to the changes produced by the training, improving the stretch tolerance, and producing morphological and neurological adaptations [76]. The compensatory effects of training, against the tendency to shorten shown by the hamstring, could be the cause of the absence of statistical differences between maturation groups. 

When kinanthropometric variables were compared, significant differences were found in body mass, height, and sitting height in males. Females showed statistical differences in body mass and height. The maturation process seemed to have a statistically significant relationship with the kinanthropometric variables, as long as the early maturers showed higher values in all the variables. The differences shown in body mass and height could be related to the changes in hormone concentration around the APHV [10]. Along this line, sex steroids, whose concentration increases during the maturation process [66], play an important role in the fat and lean mass accumulation [77] and could be the cause of the differences observed in body mass. Moreover, growth hormone (GH) has an important influence on the maturation process [78]. An increase in the GH concentration has been observed during puberty, doubling prior basal values [79]. Height is markedly influenced by GH. Therefore, the differences observed in height between the groups could be related to the fact that GH increase is closely related in time to the PHV [79]. Furthermore, the body does not grow proportionally, and growth starts earlier in the cranial, proximal, and general structures [10,11]. As a consequence, early maturers showed higher sitting height than late maturers. However, none of the studies analyzed the changes in females, so this is an important issue for future research.

The current study found that the maturation process seemed to have a statistical relationship with BMI in males but not in females. BMI is a variable that relates body mass and height [80]. Height is mainly influenced by GH during the growing period in both sexes [79]. In spite of this, body mass could not differentiate between fat, muscle, bone, skin and residual masses [80] and, although all of them increased during the growing period [81,82], these changes depend on sex. Along this line, males showed a higher increase in muscle mass as a consequence of testosterone changes [66]. Muscle mass weighs more than the other masses [83,84], which could induce a higher increase in body mass which could not be compensated with the increase in height in males. Despite these promising results, questions remain. 

Another important result was that the maturational process was shown to have an influence on fat percentage in males [29,30,85]. None of the studies analyzed changes in females. The differences found between the early and on-time groups, and on-time and late groups could be related to the positive relationship between an increased amount of adipose tissue and an earlier and shorter maturational process [29,30]. Furthermore, the interaction between the sex hormones and the GH/Insulin-like Growth Factor-I axis seemed to be the prime determinants of changing body composition during adolescence, so changes around PHV could be influencing the differences found in fat mass [81,82]. Despite these promising results, questions remain about the changes depending on sex.

The articles included in the meta-analysis showed a moderate to high heterogeneity in both the kinanthropometric and physical fitness variables (Figure 2 and Figure 3). This heterogeneity could be due to the differences between the methods used to assess these variables. All the tests used to assess physical fitness were widely used, reliable, and valid tests [73,86,87,88,89,90,91]. There was strong evidence of the positive effects of warming up on sports performance [92]. However, only five articles indicated warming-up before the physical fitness test [9,32,34,42,58], and only López-Plaza et al. [9] described it in detail. The differences in the warm-up protocols could be a risk of bias in the results of physical fitness tests. Another concern about the measurement protocol was the order of the tests performed. Only Matthys et al. [42] provided a specified order in the administration of the tests. It has been shown that the order of the physical tests is important for the final results, as the fatigue caused by the different tests can have an influence on the performance of the latter assessments [93]. The protocols used to assess the kinanthropometric variables also varied among the articles included. The standardized protocols described by Lohman et al. (1988) [42,55,57,59,60], the International Society for the Advancement in Kinanthropometry [9], and the International Working Group on Kinanthropometry [31] were used, and six of the articles did not specify the method followed for the measurements [32,33,34,54,56,58]. Despite it being a valid and reliable method, kinanthropometry assessment can be negatively affected by external factors, such as the methodology used or the researcher’s experience [15,16]. It has been shown that a percentage of error of 11% can be introduced in the results due to the protocol and the researcher’s training [15,16]. Moreover, four estimation formulas were used to assess the fat mass percentage (Table 1). There is evidence that shows that the results of fat mass obtained with different equations are not interchangeable nor comparable [94]. This lack of agreement in the methods used could be affecting the results shown in the different articles included in the meta-analysis. On the other hand, there was no agreement on the method used to assess the maturation offset, using up to four different methods to assess the biological maturation (radiography, anthropometry, development of the secondary sexual characteristics, and age at menarche). Furthermore, even in the articles that assess the maturation with the same method, different protocols are used, which could be a potential risk of bias of the results obtained in the meta-analysis. Most of the studies used hand and wrist radiography methods, considered the gold standard of the maturity status assessment [32,34,55,57,60], or regression equations validated in broad samples of both sexes [9,31,33,42,56,58]. Two studies used the secondary sexual characteristic methods [54,59], which could increase the risk of bias due to the subjectivity of the procedures [11]. Moreover, Hammami et al. [58] and Sogut et al. [34] only divided their samples into two groups, instead of distinguishing between early, on-time, and late maturers, as proposed in previous research studies [10] and accepted in the majority of studies. This difference in the classification of the subjects could provoke a loss of information in relation to those subjects whose maturational status is ±1 year from APHV with respect to their late or early maturer peers [10].

Throughout the analysis, some limitations in the current literature were observed. The most remarkable was the lack of studies performed with female populations. Only two studies were found that analyzed the relationship of biological maturation with the kinanthropometric and physical fitness variables in females [34,54]. Thus, due to the variables included, only the height, body mass, and BMI could be included in the meta-analysis, and none of the physical fitness tests met the inclusion criteria because of the lack of agreement in the assessment [34,54]. Another prominent limitation of the present literature was that muscle mass, bone mass, and proportionality information was not provided in the included research studies. The influence of muscle mass on sports performance and the development that occurs during maturation have been extensively documented [23,62,63,84]. Furthermore, the bone mass plays an important role in sports performance, as the structural support of the plastic components [28]; or in the body proportionality variables, where studies on various sports have shown that certain bone proportions could favor the performance in specific sports such as Olympic weightlifting, swimming or combat sports [27,95,96]. 

There are some practical applications derived from the results obtained. The relationships shown in this meta-analysis between the CMJ, SJ, handgrip strength test, medicinal ball throw, and agility *t*-test results with the maturation process make it necessary to take into account the biological maturation of the young athletes when these tests are used in a talent identification process. However, other tests such as the Yo-Yo test or the sit and reach test seem to have less relation with the maturity of the athletes, which may indicate that the results in these tests could be compared regardless of the maturation process. Furthermore, the results of the fitness tests are expressed in absolute values and could have been influenced by the changes in the kinanthropometric variables. Relative measures according to different kinanthropometric variables could help to clarify this relationship.

Due to this, future lines of research could aim to improve the evidence of the relationship of biological maturation with kinanthropometric and physical fitness in females; to analyze the influence of biological maturation on kinanthropometry and physical test with longitudinal designs; to clarify the effect of the changes in kinanthropometric measurements on the physical fitness performance; to relativize the fitness test results related to biological maturation according to different kinanthropometric variables, specifically in those kinanthropometric variables that change during puberty and may influence the result of the fitness test; to investigate the relationship of biological maturation with muscle mass and the relationship with physical performance; and to improve the knowledge about the evolution of proportionality variables during the maturation process, and the relationship with physical and sports performance.

## 5. Conclusions

Biological maturation seems to have a significant relationship with the kinanthropometric and physical fitness variables in males. Early maturers showed higher values of body mass, height, BMI, and fat mass percentage, and also showed better results in physical fitness tests, with marked differences in medicine ball throw, handgrip strength, CMJ, and SJ, tests that were dependent on strength and the production of power, probably as a result of the changes in the hormonal environment and the effect on muscle gain. However, maturational status seems not to have a relationship with the Yo-Yo test and sit and reach results. Few studies were found with females, and differences were only found between early and late maturers in body mass and height, so more research would be necessary. The relationships shown in this meta-analysis between the strength-dependent fitness test with the maturation process make it necessary to assess the biological maturation when these tests aim to help in a talent identification process. In spite of this, more research is needed to clarify the relationship of maturation with physical fitness and kinanthropometric variables in female populations and the changes in the muscle and bone variables during the maturation processes of both sexes.

## Figures and Tables

**Table 1:** Data extraction of the selected studies.

Author	Sex (n)	Age (X ± SD)	Competitive Level	Sport	Maturation Offset Estimation	Maturational Status	Physical Fitness Tests	Kinanthropometric Measurements and Body Composition
Arede et al. (2018) [33]	M (34)	14.6 ± 0.23	National	Basketball	Method: kinanthropometry; Equation: Mirwald et al. (2002)	Pre-pubertal, pubertal, post-pubertal	SJ, CMJ, ABK, medicinal ball throw (2 kg), sprint (20 m), Yo-Yo intermittent recovery test, agility T-test, sit and reach test	Body mass, height, sitting height
Carling et al. (2012) [61]	M (158)	13.5 ± 0.4	Elite	Football	Method: Hand and wrist radiography; Greulich and Pyle (1959)	Delayed, average, advanced	CMJ, sprint (10, 20, and 40 m), VO2máx, quadriceps isokinetic strength	Body mass, height, four skinfolds (triceps, biceps, subscapular, iliac crest), fat mass percentage (method: kinanthropometry; equation: Durnin and Womersley)
Figueiredo et al. (2009) [55]	M (87)	11.0 to 12.0 (11.8 ± 0.53)	Regional	Football	Method: Hand and wrist radiography; Roche (1988) and Tanner (1962)	Late, on time, early	SJ, CMJ, Yo-Yo intermittent recovery test, seven-sprint protocol, agility shuttle run	Body mass, height, sitting height, four skinfolds (triceps, subscapular, iliac crest, calf)
	M (72)	13.0 to 14.9 (14.4 ± 0.56)						
Gastin et al. (2013) [56]	M (52)	-	Regional	Australian Football	Method: kinanthropometry; Equation: Mirwald et al. (2002)	Less mature, more mature	20 m shuttle run test, sprint (20 m)	Body mass, height, sitting height
Gouvea et al. (2016) [57]	M (28)	12.8 ± 1.2	Semi-professional	Football	Method: Hand and wrist radiography; Greulich and Pyle (1959)	Late, on time, early	SJ, CMJ, handgrip strength, sit and reach test, sit-up test, Yo-Yo intermittent recovery test	Body mass, height, fat mass percentage (Method: Bod Pod; equation: Lohman), BMI
Guimaraes et al. (2019) [31]	M (152)	13.3 ± 0.7	Regional	Basketball	Method: kinanthropometry; Equation: Mirwald et al. (2002)	Late, average, early	SJ, CMJ, medicine ball throw (3kg), Yo-Yo intermittent recovery test, sprint (20 m), agility T-test, handgrip strength	Body mass, height, sitting height, leg length
Hammami et al. (2017) [58]	M (56)	12 to 14	Elite	Handball	Method: kinanthropometry; Equation: Moore et al. (2015)	Pre-PHV, post-PHV	SJ, CMJ, SLJ, H-CMJ, H-SJ, 3HOPT, sprint (10, 20, 30 m), agility T-half test, CODAT	Body mass, height, fat mass percentage (method: kinanthropometry; equation: Slaughter)
Leonardi et al. (2018) [54]	F (47)	13.5 (11.5–15.6)	Regional	Basketball	Method: sexual maturation; Age at menarche	Late, average, early	CMJ, Yo-Yo intermittent recovery test, Line Drill test	Body mass, height, BMI, four skinfolds (triceps, subscapular, iliac crest, calf)
López-Plaza et al. (2016) [9]	M (89)	13.7 ± 0.6	Elite	Kayak	Method: kinanthropometry; Equation: Mirwald et al. (2002)	Pre-PHV, circum-PHV, post-PHV	SJ, CMJ, medicine ball throw (3 kg), VO2max	Body mass, height, sitting height, BMI, six skinfolds (triceps, subscapular, biceps, iliac crest, supraspinal, calf), fat mass percentage (method: kinanthropometry; equation: Slaughter), muscle mass percentage (method: kinanthropometry; equation: Poortmans)
	M (82)	13.7 ± 0.6	Elite	Canoe				
Matta et al. (2014) [59]	M (114)	14.2 ± 0.5	Regional	Football	Method: sexual maturation; Marshall and Tanner (1962)	Sexual maturation P3, P4, P5	SJ, CMJ, Yo-Yo intermittent recovery test, RAST, sprint (5, 30 m), agility *t*-test	Body mass, height, four skinfolds (triceps, subscapular, iliac crest, calf)
Matthys et al. (2012) [42]	M (168)	14.5±0.13	Regional and national	Handball	Method: kinanthropometry; Equation: Mirwald et al. (2002)	Late, on time, early	5-jump test, handgrip strength, sprint (5, 20 m)	Body mass, height, sitting height, body fat percentage (Method: electric bioimpedance)
Sogut et al. (2019) [34]	F (61)	11.8±0.8	National	Tennis	Method: Hand and wrist radiography; Lohman and Roche (1988)	Latest, Earliest	Handgrip strength, hexagon agility test	Body mass, height, sitting height, two skinfolds (triceps, calf), fat mass percentage (method: kinanthropometry; equation: Slaughter)
Valente-Dos Santos et al. (2014) [60]	M (36)	12	Regional	Football	Method: Hand and wrist radiography; Roche (1988) Malina (2004)	Late, on time, early	CMJ, agility shuttle run test, dribbling speed	Body mass, height, two skinfolds (triceps, subscapular), fat mass (method: kinanthropometry; equation: Slaughter), fat free mass
	M (53)	13						
	M (91)	14						
	M (51)	15						

X: mean; SD: standard deviation; M: males; F: females; P3: maturation stage occurring around 12 years old (females) and 13 years old (males); P4: maturation stage occurring around 13 years old (females) and 14 years old (males); P5: maturation stage occurring around 15 years old (males and females); SJ: squat jump; CMJ: countermovement jump; ABK: abalakov jump; SLJ: squat long jump; H-CMJ: horizontal countermovement jump; H-SJ: horizontal squat jump; 3HOPT: three hops test; CODAT: change of direction and acceleration test; RAST: running-based anaerobic sprint test.

**Table 2:** Quality assessment of the selected studies.

Study	1	2	3	4	5	6	7	8	9	10	11	12	13	14	15	16	17	18	19	20	21	22	100%	Total
Arede et al. (2018) [33]	1	1	1	1	1	0	0	1	0	0	1	1	0	1	1	1	1	1	0	1	0	1	68.18	15
Carling et al. (2012) [32]	1	1	1	1	1	0	1	1	0	0	1	1	1	1	1	1	1	1	0	1	1	0	77.27	17
Figueiredo et al. (2009) [55]	1	1	1	1	1	0	1	1	0	0	1	0	1	1	1	1	1	1	0	1	0	0	68.18	15
Gastin et al. (2013) [56]	1	1	1	1	1	0	1	1	0	0	1	1	1	1	1	1	1	1	0	1	0	1	77.27	17
Gouvea et al. (2016) [57]	1	1	1	1	1	0	1	1	0	0	1	1	1	1	1	1	1	1	1	1	0	1	81.82	18
Guimaraes et al. (2019) [31]	1	1	1	1	1	1	1	1	0	0	1	1	1	1	1	1	1	1	1	1	1	1	90.91	20
Hammami et al. (2017) [58]	1	1	1	1	0	0	1	0	0	0	1	1	1	1	1	1	1	1	1	1	1	1	77.27	17
Leonardi et al. (2018) [54]	1	1	1	1	1	0	1	1	0	0	1	1	1	1	1	1	1	1	0	1	0	1	77.27	17
López-Plaza et al. (2016) [9]	1	1	1	1	1	1	1	1	0	0	1	1	1	1	1	1	1	1	0	1	1	1	86.36	19
Matta et al. (2014) [59]	1	1	1	1	1	0	1	1	0	0	1	1	1	1	1	1	1	1	0	1	0	0	72.73	16
Matthys et al. (2012) [42]	1	1	1	1	1	0	1	1	0	0	1	1	1	1	1	1	1	1	1	1	0	1	81.82	18
Sogut et al. (2019) [34]	1	1	1	1	1	1	1	1	0	0	1	1	1	1	1	1	1	1	1	1	0	1	86.36	19
Valente-Dos Santos et al. (2014) [60]	1	1	1	1	1	0	1	1	0	0	1	1	1	1	1	1	1	1	1	1	1	1	86.36	19
Mean of total scores:																							79.37	17.46

**Table 3:** Means and statistical differences between groups in physical fitness tests and kinanthropometric variables.

	Number of Studies	Early (Mean ± SD)	On Time (Mean ± SD)	Late (Mean ± SD)	Early vs. On Time				Early vs. Late				On Time vs. Late			
					SMD	95% CI	Z	*p*	SMD	95% CI	Z	*p*	SMD	95% CI	Z	*p*
CMJ (m)	8 (Table S1)	0.32 ± 0.05	0.31 ± 0.05	0.30 ± 0.05	0.17	0.01 to 0.33	2.06	0.04	0.38	0.04 to 0.73	2.18	0.03	0.12	−0.07 to 0.31	1.20	0.23
SJ (m)	6 (Table S2)	0.28 ± 0.04	0.27 ± 0.05	0.26 ± 0.06	0.23	0.01 to 0.45	2.08	0.04	0.35	−0.21 to 0.92	1.22	0.22	0.04	−0.26 to 0.33	0.24	0.81
Medicine ball throw (m)	3 (Table S6)	5.96 ± 0.94	5.19 ± 0.71	4.60 ± 0.76	0.99	0.73 to 1.25	7.40	<0.001	1.58	0.94 to 2.23	4.83	<0.001	0.89	0.60 to 1.18	6.04	<0.001
Handgrip strength (kg)	3 (Table S5)	42.50 ± 7.60	33.75 ± 6.35	26.40 ± 5.85	1.31	0.96 to 1.67	7.20	<0.001	2.31	1.79 to 2.84	8.70	<0.001	1.09	0.79 to 1.39	7.12	<0.001
Sprint 20 m (s)	6 (Table S3)	3.24 ± 0.23	3.39 ± 0.20	3.43 ± 0.22	−0.52	−1.07 to 0.04	1.81	0.07	−0.94	−1.54 to −0.34	3.08	<0.001	−0.42	−0.82 to 0.01	1.93	0.05
Yo-Yo test (m)	6 (Table S4)	1150.41 ± 488.92	1243.48 ± 487.34	1154.44 ± 374.73	−0.21	−0.54 to 0.12	1.22	0.22	0.05	−1.08 to 0.08	1.69	0.09	−0.16	−0.37 to 0.05	1.47	0.14
Agility *t*-test (s)	3 (Table S7)	9.99 ± 0.64	10.10 ± 0.56	10.20 ± 0.50	−0.19	−0.82 to 0.43	0.61	0.54	−0.52	−0.83 to −0.20	3.18	0.001	−0.13	−0.42 to 0.15	0.90	0.37
Sit and reach (m)	4 (Table S8)	0.12 ± 0.08	0.11 ± 0.09	0.11 ± 0.07	0.23	−0.08 to 0.54	1.45	0.15	0.31	−0.08 to 0.70	1.56	0.12	0.02	−0.35 to 0.38	0.09	0.93
Body mass (kg)	11 (Table S10) M	63.47 ± 8.47	54.37 ± 8.00	45.96 ± 7.26	1.07	0.77 to 1.38	6.84	<0.001	2.26	1.49 to 3.02	5.81	<0.001	1.29	0.99 to 1.59	8.41	<0.001
Height (m)	11 (Table S9) M	1.69 ± 0.06	1.63 ± 0.07	1.56 ± 0.13	0.90	0.50 to 1.29	4.44	<0.001	2.09	1.37 to 2.81	5.67	<0.001	1.13	0.76 to 1.50	6.04	<0.001
Sitting height (m)	3 (Table S12) M	86.15 ± 2.96	81.42 ± 2.85	79.91 ± 3.23	1.64	0.92 to 2.36	4.46	<0.001	2.56	1.22 to 3.91	3.74	<0.001	1.76	0.77 to 2.75	3.47	<0.001
BMI (kg/m^2^)	1 (Table S13) M	21.93 ± 2.59	20.52 ± 2.38	18.76 ± 1.73	0.54	0.20 to 0.88	3.13	0.002	1.35	0.65 to 2.05	3.80	<0.001	0.82	0.36 to 1.28	3.52	<0.001
Fat mass (%)	5 (Table S11) M	15.76 ± 5.32	13.75 ± 4.60	12.49 ± 4.77	0.37	0.06 to 0.67	2.33	0.02	0.52	−0.06 to 1.09	1.77	0.07	0.47	0.22 to 0.72	3.65	<0.001
Body mass (kg)	2 (Table S15) F	54.8 ± 10.10	−	44.75 ± 8.80	−	−	−	−	0.96	0.54 to 1.39	4.42	<0.001	−	−	−	−
Height (m)	2 (Table S14) F	1.62 ± 0.06	−	1.53 ± 0.08	−	−	−	−	1.19	0.75 to 1.63	5.31	<0.001	−	−	−	−
BMI (kg/m^2^)	2 (Table S16) F	20.5 ± 3.27	−	18.65 ± 2.54	−	−	−	−	0.56	−0.21 to 1.32	1.43	0.15	−	−	−	−

SJ: Squat jump; CMJ: Countermovement jump; BMI: Body mass index; SMD: Standardized mean differences.

## Data Availability

The datasets generated and analyzed for this study can be requested by correspondence authors in rvaquero@ucam.edu and ngonzalez@ucam.edu.

## Supplementary Materials

The following are available online at https://www.mdpi.com/1660-4601/18/1/328/s1, Figure S1: Funnel plot for the CMJ, height, and body mass variables. Table S1: Comparison between maturational groups for countermovement jump. Table S2: Comparison between maturational groups for squat jump. Table S3: Comparison between maturational groups for sprint test. Table S4: Comparison between maturational groups for Yo-Yo test. Table S5: Comparison between maturational groups for handgrip strength. Table S6: Comparison between maturational groups for medicinal ball throw. Table S7: Comparison between maturational groups for T-test. Table S8: Comparison between maturational groups for sit and reach test. Table S9: Comparison between maturational groups for height. Table S10: Comparison between maturational groups for body mass. Table S11: Comparison between maturational groups fat mass percentage. Table S12: Comparison between maturational groups for sitting height. Table S13: Comparison between maturational groups for BMI. Table S14: Comparison between early and late maturers groups for height. Table S15: Comparison between early and late maturers groups for body mass. Table S16: Comparison between early and late maturers groups for BMI.
