# Supplementary material for: Relationship between Biological Maturation, Physical Fitness, and Kinanthropometric Variables of Young Athletes: A Systematic Review and Meta-Analysis
Source: Int J Environ Res Public Health. 2021 Jan 5;18(1):328. doi: 10.3390/ijerph18010328 (PMC7795393; doi:10.3390/ijerph18010328)
Supplement: Supplementary file 1 [file ijerph-18-00328-s001.pdf]

SUPPLEMENTARY FIGURES AND TABLES

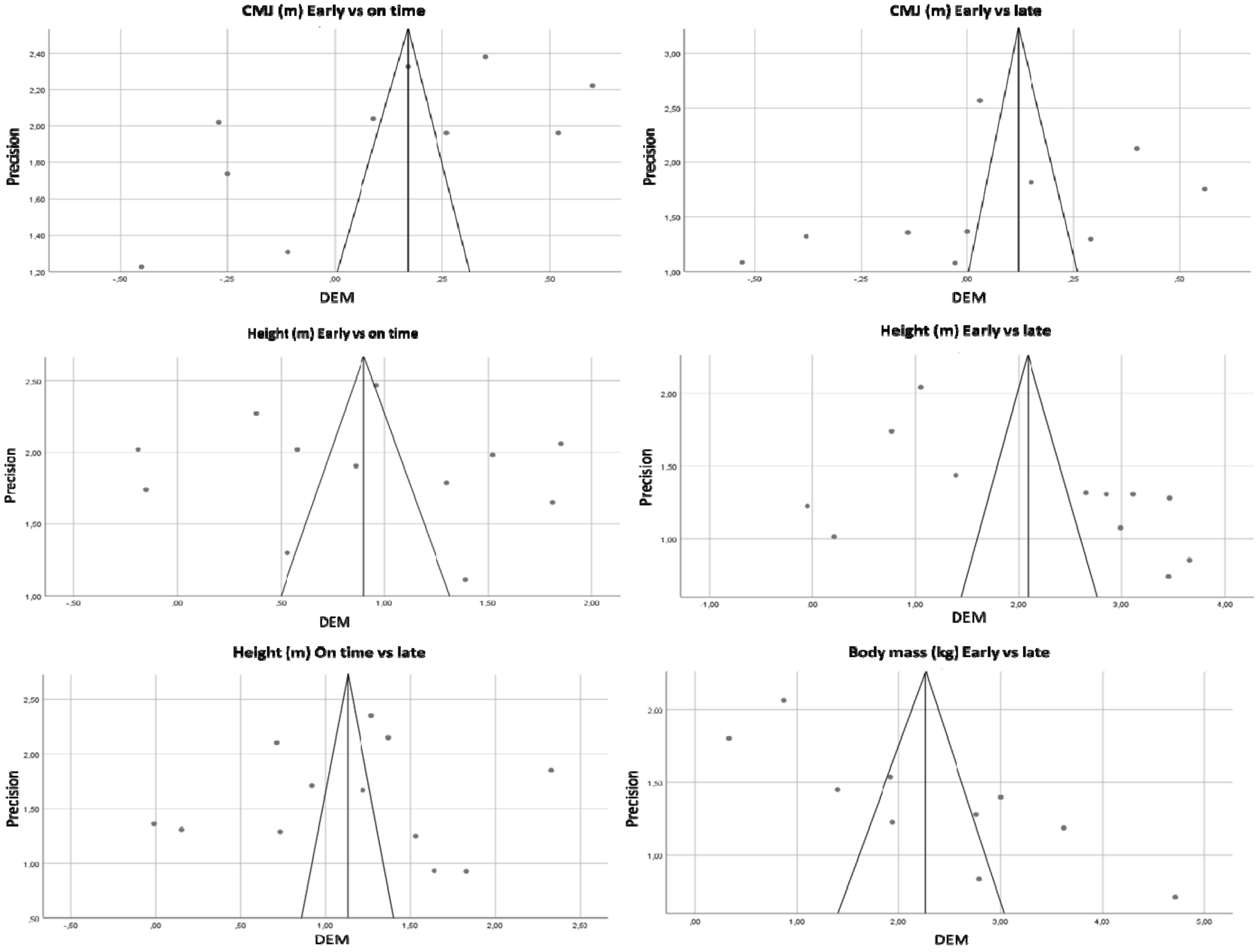

Figure S1. Funnel plot for the CMJ, height and body mass variables.

Fitness tests: Male

Table S1. Comparison between maturational groups for counter movement jump (CMJ) (cm).

	Authors	Group A		Group B		SMD	95% CI		z	p	Specific weight (%)
		N	Mean±SD	N	Mean±SD		Min	Max			
Early (Group A) and On Time (Group B)	Arede et al. (2018)	15	30.04±5.00	10	32.04±3.04	−0.45	−1.26	0.37	1.08	0.281	4.0
	Figueiredo et al. (2009)—1	25	26.30±5.20	45	25.80±5.50	0.09	−0.40	0.58	0.37	0.713	11.0
	Figueiredo et al. (2009)—2	23	33.90±3.90	45	31.50±4.90	0.52	0.01	1.03	1.99	0.047	10.1
	Guimaraes et al. (2019)	30	31.60±6.00	84	29.50±6.00	0.35	−0.07	0.77	1.62	0.104	14.9
	López-Plaza et al. (2016)—1	44	38.00±7.00	36	34.00±6.00	0.60	0.15	1.05	2.62	0.008	12.9
	López-Plaza et al. (2016)—2	30	35.00±8.00	30	33.00±7.00	0.26	−0.25	0.77	1.01	0.311	10.1
	Matta et al. (2014)	41	31.10±5.50	41	30.20±5.00	0.17	−0.26	0.60	0.77	0.443	13.9
	Valente-Dos Santos et al. (2014)—2	8	28.40±3.50	37	29.00±5.70	−0.11	−0.87	0.66	0.28	0.779	4.5
	Valente-Dos Santos et al. (2014)—3	21	30.20±5.80	62	31.80±5.80	−0.27	−0.77	0.22	1.08	0.280	10.6
	Valente-Dos Santos et al. (2014)—4	21	32.90±3.60	27	34.00±4.90	−0.25	−0.82	0.33	0.85	0.398	8.0
	Pooled standardized mean differences:					0.17	0.01	0.33	2.06	0.038	100
	Authors	Group A		Group C		SMD	95% CI		z	p	Specific weight (%)
		N	Mean±SD	N	Mean±SD		Min	Max			
Early (Group A) and Late (Group C)	Arede et al. (2018)	15	30.04±5.00	9	34.70±6.14	−0.83	−1.69	0.04	1.87	0.061	8.7
	Figueiredo et al. (2009)—1	25	26.30±5.20	17	22.70±5.40	0.67	0.03	1.30	2.07	0.038	11.6
	Guimaraes et al. (2019)	30	31.60±6.00	36	29.30±5.30	0.40	−0.09	0.89	1.62	0.106	13.8
	Hammami et al. (2017)	22	30.42±5.39	34	24.76±3.86	1.24	0.65	1.82	4.13	<0.001	12.3
	López-Plaza et al. (2016)—1	44	38.00±7.00	9	34.00±8.00	0.55	−0.18	1.28	1.49	0.137	10.3
	López-Plaza et al. (2016)—2	30	35.00±8.00	22	32.00±6.00	0.41	−0.15	0.97	1.44	0.149	12.7
	Matta et al. (2014)	41	31.10±5.50	32	28.30±4.30	0.55	0.08	1.02	2.30	0.021	14.1
	Valente-Dos Santos et al. (2014)—2	8	28.40±3.50	8	27.40±3.50	0.27	−0.72	1.26	0.54	0.591	7.4
	Valente-Dos Santos et al. (2014)—3	21	30.20±5.80	8	32.60±5.40	−0.41	−1.23	0.41	0.98	0.329	9.1
	Pooled standardized mean differences:					0.38	0.04	0.73	2.18	0.029	100
	Authors	Group B		Group C		SMD	95% CI		z	p	Specific weight (%)
		N	Mean±SD	N	Mean±SD		Min	Max			
On time (Group B) and Late (Group C)	Arede et al. (2018)	10	32.04±3.04	9	34.70±6.14	−0.53	−1.45	0.39	1.14	0.255	4.4
	Figueiredo et al. (2009)—1	45	25.80±5.50	17	22.70±5.40	0.56	−0.01	1.13	1.93	0.053	11.5
	Gouvea et al. (2016)	18	28.40±3.60	6	28.50±3.90	−0.03	−0.95	0.90	0.06	0.955	4.3
	Guimaraes et al. (2019)	84	29.50±6.00	36	29.30±5.30	0.03	−0.36	0.42	0.17	0.863	24.2
	López-Plaza et al. (2016)—1	36	34.00±6.00	9	34.00±8.00	0.00	−0.73	0.73	0.00	1.000	6.9
	López-Plaza et al. (2016)—2	30	33.00±7.00	22	32.00±6.00	0.15	−0.40	0.70	0.53	0.595	12.2
	Matta et al. (2014)	41	30.20±5.00	32	28.30±4.30	0.40	−0.07	0.87	1.68	0.093	16.9
	Valente-Dos Santos et al. (2014)—1	22	26.50±5.10	10	28.30±3.00	−0.38	−1.14	0.37	1.00	0.318	6.5

Valente-Dos Santos et al. (2014)—2	37	29.00±5.70	8	27.40±3.50	0.29	−0.48	1.06	0.74	0.457	6.3
Valente-Dos Santos et al. (2014)—3	62	31.80±5.80	8	32.60±5.40	−0.14	−0.87	0.60	0.37	0.714	6.8
Pooled standardized mean differences:					0.12	-0.07	0.31	1.20	0.229	100

CMJ: Counter movement jump; Figueiredo et al. (2009)—1: 11.0 to 12.9 years-old; Figueiredo et al. (2009)—2: 13.0 to 14.9 years-old; López-Plaza et al. (2016)—1: kayakers; López-Plaza et al. (2016)—2: canoeists; Valente-Dos Santos et al. (2014)—1: 12 years-old; Valente-Dos Santos et al. (2014)—2: 13 years-old; Valente-Dos Santos et al. (2014)—3: 14 years-old; Valente-Dos Santos et al. (2014)—4: 15 years-old.

Table S2. Comparison between maturational groups for squat jump (SJ) (cm).

	Authors	Group A		Group B		SMD	95% CI		z	p	Specific weight (%)
		N	Mean±SD	N	Mean±SD		Min	Max			
Early (Group A) and On Time (Group B)	Arede et al. (2018)	15	30.91±2.07	10	30.83±4.76	0.02	-0.78	0.82	0.06	0.955	7.6
	Figueiredo et al. (2009) - 1	25	24.90±5.60	45	24.70±5.10	0.04	-0.45	0.53	0.15	0.880	20.3
	Figueiredo et al. (2009) - 2	23	30.60±4.00	45	28.40±3.70	0.57	0.06	1.08	2.19	0.028	18.5
	Guimaraes et al. (2019)	30	25.50±6.20	84	24.70±5.80	0.13	-0.28	0.55	0.63	0.527	27.9
	Matta et al. (2014)	41	27.80±4.50	41	26.40±4.30	0.32	-0.12	0.75	1.42	0.156	25.6
	Pooled standardized mean differences:					0.23	0.01	0.45	2.08	0.037	100

	Authors	Group A		Group C		SMD	95% CI		z	p	Specific weight (%)
		N	Mean±SD	N	Mean±SD		Min	Max			
Early (Group A) and Late (Group C)	Arede et al. (2018)	15	30.91±2.07	9	33.02±6.56	-0.48	-1.31	0.36	1.11	0.267	16.5
	Figueiredo et al. (2009) - 1	25	24.90±5.60	17	23.30±6.30	0.27	-0.35	0.89	0.84	0.398	19.7
	Guimaraes et al. (2019)	30	25.50±6.20	36	26.30±6.90	-0.12	-0.60	0.37	0.48	0.627	21.7
	Hammami et al. (2017)	22	29.05±3.88	34	23.88±3.88	1.31	0.72	1.91	4.34	<0.001	20.1
	Matta et al. (2014)	41	27.80±4.50	32	24.90±4.50	0.64	0.16	1.11	2.63	0.008	21.9
	Pooled standardized mean differences:					0.35	-0.21	0.92	1.22	0.221	100

	Authors	Group B		Group C		SMD	95% CI		z	p	Specific weight (%)
		N	Mean±SD	N	Mean±SD		Min	Max			
On time (Group B) and Late (Group C)	Arede et al. (2018)	10	30.83±4.76	9	33.02±6.56	-0.37	-1.28	0.54	0.79	0.427	9.2
	Figueiredo et al. (2009) - 1	45	24.70±5.10	17	23.30±6.30	0.25	-0.31	0.81	0.89	0.374	20.8
	Gouvea et al. (2016)	18	29.20±4.70	6	28.50±3.90	0.15	-0.78	1.07	0.32	0.751	8.9
	Guimaraes et al. (2019)	84	24.70±5.80	36	26.30±6.90	-0.26	-0.65	0.13	1.29	0.195	34.0
	Matta et al. (2014)	41	26.40±4.30	32	24.90±4.50	0.34	-0.13	0.80	1.42	0.154	27.1
	Pooled standardized mean differences:					0.04	-0.26	0.33	0.24	0.809	100

SJ: Squat jump; Figueiredo et al. (2009) – 1: 11.0 to 12.9 years-old; Figueiredo et al. (2009) – 2: 13.0 to 14.9 years-old.

Table S3. Comparison between maturational groups for 20 meters sprint test (s).

Comparison	Authors	Group A		Group B		SMD	95% CI		z	p	Specific weight (%)
		N	Mean±SD	N	Mean±SD		Min	Max			
Early (Group A) and On Time (Group B)	Arede et al. (2018)	15	3.17±0.13	10	3.11±0.12	0.46	-0.35	1.27	1.11	0.267	19.5
	Carling et al. (2012)	34	3.23±0.12	98	3.34±0.13	-0.86	-1.26	-0.45	4.16	<0.001	28.3
	Guimaraes et al. (2019)	30	3.40±0.20	84	3.70±0.30	-1.07	-1.51	-0.63	4.78	<0.001	27.5
	Matthys et al. (2012)	13	3.36±0.22	135	3.42±0.22	-0.27	-0.84	0.30	0.93	0.350	24.7
	Pooled standardized mean differences:					-0.52	-1.07	0.04	1.81	0.069	100

Comparison	Authors	Group A		Group C		SMD	95% CI		z	p	Specific weight (%)
		N	Mean±SD	N	Mean±SD		Min	Max			
Early (Group A) and Late (Group C)	Arede et al. (2018)	15	3.17±0.13	9	3.13±0.14	0.29	-0.54	1.12	0.68	0.495	14.8
	Carling et al. (2012)	34	3.23±0.12	26	3.44±0.11	-1.79	-2.40	-1.18	5.76	<0.001	17.1
	Gastin et al. (2013)	25	3.50±0.40	25	3.60±0.40	-0.25	-0.80	0.31	0.87	0.386	17.6
	Guimaraes et al. (2019)	30	3.40±0.20	36	3.70±0.20	-1.48	-2.03	-0.93	5.28	<0.001	17.6
	Hammami et al. (2017)	22	2.80±0.30	34	3.16±0.25	-1.31	-1.90	-0.72	4.34	<0.001	17.2
	Matthys et al. (2012)	13	3.36±0.22	20	3.56±0.20	-0.94	-1.68	-0.20	2.49	0.012	15.7
	Pooled standardized mean differences:					-0.94	-1.54	-0.34	3.08	0.002	100

Comparison	Authors	Group B		Group C		SMD	95% CI		z	p	Specific weight (%)
		N	Mean±SD	N	Mean±SD		Min	Max			
On time (Group B) and Late (Group C)	Arede et al. (2018)	10	3.11±0.12	9	3.13±0.14	-0.15	-1.05	0.75	0.32	0.749	14.3
	Carling et al. (2012)	98	3.34±0.13	26	3.44±0.11	-0.79	-1.23	-0.34	3.48	<0.001	28.3
	Guimaraes et al. (2019)	84	3.70±0.30	36	3.70±0.20	0.00	-0.39	0.39	0.00	1.000	30.4
	Matthys et al. (2012)	135	3.42±0.22	20	3.56±0.20	-0.64	-1.12	-0.17	2.64	0.008	27.0
	Pooled standardized mean differences:					-0.42	-0.84	0.01	1.93	0.053	100

Table S4. Comparison between maturational groups for Yo-Yo test (m).

Comparison	Authors	Group A		Group B		SMD	95% CI		z	p	Specific weight (%)
		N	Mean±SD	N	Mean±SD		Min	Max			
Early (Group A) and On Time (Group B)	Arede et al. (2018)	15	817.14±328.62	10	1062.22±371.54	-0.68	-1.51	0.14	1.62	0.104	8.8
	Figueiredo et al. (2009) - 1	25	1208.00±788.00	45	1308.00±657.00	-0.14	-0.63	0.35	0.56	0.574	13.6
	Figueiredo et al. (2009) - 2	23	2617.00±902.00	45	2478.00±935.00	0.15	-0.35	0.65	0.58	0.562	13.4
	Guimaraes et al. (2019)	30	882.70±362.20	84	728.60±370.20	0.42	0.00	0.84	1.94	0.052	14.8
	Matta et al. (2014)	41	682.40±380.50	41	639.00±395.00	0.11	-0.32	0.54	0.50	0.616	14.6
	Valente-Dos Santos et al. (2014) - 2	8	820.00±420.00	37	1044.00±332.00	-0.63	-1.41	0.14	1.60	0.110	9.4
	Valente-Dos Santos et al. (2014) - 3	21	950.00±334.00	62	1286.00±402.00	-0.86	-1.37	-0.35	3.29	<0.001	13.2
	Valente-Dos Santos et al. (2014) - 4	21	1226.00±396.00	27	1402.00±436.00	-0.41	-0.99	0.16	1.40	0.160	12.2
	Pooled standardized mean differences:					-0.21	-0.54	a 0.12	1.22	0.222	100
	Authors	Group A		Group C		SMD	95% CI		z	p	Specific weight (%)
		N	Mean±SD	N	Mean±SD		Min	Max			
Early (Group A) and Late (Group C)	Arede et al. (2018)	15	817.14±328.62	9	1164.44±194.37	-1.17	-2.07	-0.27	2.54	0.011	14.7
	Figueiredo et al. (2009) - 1	25	1208.00±788.00	17	1774.00±725.00	-0.73	-1.36	-0.09	2.24	0.025	18.0
	Guimaraes et al. (2019)	30	882.70±362.20	36	712.20±277.10	0.53	0.04	1.02	2.10	0.035	19.7
	Matta et al. (2014)	41	682.40±380.50	32	720.00±469.90	-0.09	-0.55	0.37	0.37	0.708	20.0
	Valente-Dos Santos et al. (2014) - 2	8	820.00±420.00	8	1382.00±286.00	-1.48	-2.62	-0.33	2.53	0.011	12.1
	Valente-Dos Santos et al. (2014) - 3	21	950.00±334.00	8	1174.00±296.00	-0.67	-1.51	0.16	1.57	0.115	15.5
	Pooled standardized mean differences:					-0.50	-1.08	0.08	1.69	0.091	100
	Authors	Group B		Group C		SMD	95% CI		z	p	Specific weight (%)
		N	Mean±SD	N	Mean±SD		Min	Max			
On time (Group B) and Late (Group C)	Arede et al. (2018)	10	1062.22±371.54	9	1164.44±194.37	-0.34	-1.25	0.57	0.73	0.464	5.6
	Figueiredo et al. (2009) - 1	45	1308.00±657.00	17	1774.00±725.00	-0.69	-1.26	-0.12	2.37	0.018	14.0
	Gouvea et al. (2016)	18	655.60±185.20	6	520.00±207.10	0.71	-0.24	1.66	1.47	0.140	5.1
	Guimaraes et al. (2019)	84	728.60±370.20	36	712.20±277.10	0.05	-0.34	0.44	0.24	0.811	30.1
	Matta et al. (2014)	41	639.00±395.00	32	720.00±469.90	-0.19	-0.65	0.27	0.80	0.424	21.4
	Valente-Dos Santos et al. (2014) - 1	22	962.00±274.00	10	978.00±416.00	-0.05	-0.80	0.70	0.13	0.896	8.2
	Valente-Dos Santos et al. (2014) - 2	37	1044.00±332.00	8	1382.00±286.00	-1.04	-1.84	-0.24	2.56	0.010	7.3
	Valente-Dos Santos et al. (2014) - 3	62	1286.00±402.00	8	1174.00±296.00	0.29	-0.45	1.02	0.76	0.448	8.4
	Pooled standardized mean differences:					-0.16	-0.37	0.05	1.47	0.141	100

Figueiredo et al. (2009) – 1: 11.0 to 12.9 years-old; Figueiredo et al. (2009) – 2: 13.0 to 14.9 years-old; Valente-Dos Santos et al. (2014) – 2: 13 years-old; Valente-Dos Santos et al. (2014) – 3: 14 years-old; Valente-Dos Santos et al. (2014) – 4: 15 years-old.

Table S5. Comparison between maturational groups for hand grip strength.

	Hand grip strength (kg)	Group A		Group B		SMD	95% CI		z	p	Specific weight (%)
		N	Mean±SD	N	Mean±SD		Min	Max			
Early (Group A) and On Time (Group B)	Guimaraes et al. (2019)	30	33.00±6.00	84	25.70±5.60	1.27	0.82	1.72	5.54	<0.001	63.4
	Matthys et al. (2012)	13	52.00±9.20	135	41.80±7.10	1.39	0.80	1.98	4.61	<0.001	36.6
	Pooled standardized mean differences:					1.31	0.96	1.67	7.20	<0.001	100

	Hand grip strength (kg)	Group A		Group C		SMD	95% CI		z	p	Specific weight (%)
		N	Mean±SD	N	Mean±SD		Min	Max			
Early (Group A) and Late (Group C)	Guimaraes et al. (2019)	30	33.00±6.00	36	21.00±4.60	2.25	1.62	2.87	7.04	<0.001	69.5
	Matthys et al. (2012)	13	52.00±9.20	20	31.80±7.10	2.47	1.53	3.41	5.13	<0.001	30.5
	Pooled standardized mean differences:					2.31	1.79	2.84	8.70	<0.001	100

	Hand grip strength (kg)	Group B		Group C		SMD	95% CI		z	p	Specific weight (%)
		N	Mean±SD	N	Mean±SD		Min	Max			
On time (Group B) and Late (Group C)	Gouvea et al. (2016)	18	28.90±7.60	6	21.20±3.30	1.08	0.10	2.07	2.16	0.030	9.3
	Guimaraes et al. (2019)	84	25.70±5.60	36	21.00±4.60	0.88	0.47	1.28	4.23	<0.001	54.2
	Matthys et al. (2012)	135	41.80±7.10	20	31.80±7.10	1.40	0.91	1.90	5.54	<0.001	36.5
	Pooled standardized mean differences:					1.09	0.79	1.39	7.12	<0.001	100

Table S6. Comparison between maturational groups for medicinal ball throw (m).

	Authors	Group A		Group B		SMD	95% CI		z	p	Specific weight (%)
		N	Mean±SD	N	Mean±SD		Min	Max			
Early (Group A) and On Time (Group B)	Arede et al. (2018)	15	6.20±0.73	10	5.96±0.33	0.38	-0.43	1.19	0.93	0.353	10.5
	Guimaraes et al. (2019)	30	4.50±0.80	84	3.60±0.60	1.36	0.91	1.81	5.87	<0.001	33.3
	López-Plaza et al. (2016) - 1	44	6.64±1.12	36	5.85±1.00	0.73	0.28	1.19	3.15	0.001	33.1
	López-Plaza et al. (2016) - 2	30	6.48±1.11	30	5.36±0.89	1.10	0.55	1.64	3.95	<0.001	23.1
	Pooled standardized mean differences:					0.99	0.73	1.25	7.40	<0.001	100
	Authors	Group A		Group C		SMD	95% CI		z	p	Specific weight (%)
		N	Mean±SD	N	Mean±SD		Min	Max			
Early (Group A) and Late (Group C)	Arede et al. (2018)	15	6.20±0.73	9	5.58±1.06	0.69	-0.16	1.55	1.59	0.111	22.3
	Guimaraes et al. (2019)	30	4.50±0.80	36	3.00±0.50	2.27	1.64	2.90	7.08	<0.001	27.2
	López-Plaza et al. (2016) - 1	44	6.64±1.12	9	5.20±0.65	1.34	0.57	2.10	3.43	<0.001	24.2
	López-Plaza et al. (2016) - 2	30	6.48±1.11	22	4.61±0.81	1.85	1.19	2.51	5.47	<0.001	26.4
	Pooled standardized mean differences:					1.58	0.94	2.23	4.83	<0.001	100
	Authors	Group B		Group C		SMD	95% CI		z	p	Specific weight (%)
		N	Mean±SD	N	Mean±SD		Min	Max			
On time (Group B) and Late (Group C)	Arede et al. (2018)	10	5.96±0.33	9	5.58±1.06	0.50	-0.42	1.41	1.06	0.288	10.0
	Guimaraes et al. (2019)	84	3.60±0.60	36	3.00±0.5	1.05	0.64	1.46	4.98	<0.001	49.4
	López-Plaza et al. (2016) - 1	36	5.85±1.00	9	5.20±0.65	0.69	-0.06	1.43	1.81	0.070	15.2
	López-Plaza et al. (2016) - 2	30	5.36±0.89	22	4.61±0.81	0.87	0.30	1.45	2.98	0.002	25.4
	Pooled standardized mean differences:					0.89	0.60	1.18	6.04	<0.001	100

López-Plaza et al. (2016) – 1: kayakers; López-Plaza et al. (2016) – 2: canoeists.

Table S7. Comparison between maturational groups for T-test (s).

Comparison	Authors	Group A N	Group A Mean±SD	Group B N	Group B Mean±SD	SMD	95% CI Min	95% CI Max	z	p	Specific weight (%)
Early (Group A) and On Time (Group B)	Arede et al. (2018)	15	10.18±0.63	10	9.88±0.37	0.53	-0.28	1.35	1.28	0.199	25.6
	Guimaraes et al. (2019)	30	9.50±0.60	84	10.00±0.70	-0.74	-1.16	-0.31	3.37	<0.001	37.3
	Matta et al. (2014)	41	10.30±0.70	41	10.40±0.60	-0.15	-0.59	0.28	0.69	0.492	37.1
	Pooled standardized mean differences:					-0.19	-0.82	0.43	0.61	0.543	100
	Authors	Group A N	Group A Mean±SD	Group C N	Group C Mean±SD	SMD	95% CI Min	95% CI Max	z	p	Specific weight (%)
Early (Group A) and Late (Group C)	Arede et al. (2018)	15	10.18±0.63	9	10.00±0.39	0.31	-0.52	1.15	0.74	0.460	14.6
	Guimaraes et al. (2019)	30	9.50±0.60	36	9.90±0.50	-0.72	-1.22	-0.22	2.82	0.004	40.3
	Matta et al. (2014)	41	10.30±0.70	32	10.70±0.60	-0.60	-1.07	-0.13	2.49	0.012	45.1
	Pooled standardized mean differences:					-0.52	-0.83	-0.20	3.18	0.001	100
	Authors	Group B N	Group B Mean±SD	Group C N	Group C Mean±SD	SMD	95% CI Min	95% CI Max	z	p	Specific weight (%)
On time (Group B) and Late (Group C)	Arede et al. (2018)	10	9.88±0.37	9	10.00±0.39	-0.30	-1.21	0.60	0.65	0.513	9.9
	Guimaraes et al. (2019)	84	10.00±0.70	36	9.90±0.50	0.15	-0.24	0.54	0.77	0.441	53.2
	Matta et al. (2014)	41	10.40±0.60	32	10.70±0.60	-0.49	-0.96	-0.03	2.06	0.038	36.9
	Pooled standardized mean differences:					-0.13	-0.42	0.15	0.90	0.369	100

Table S8. Comparison between maturational groups for sit and reach test (cm).

	Authors	Group A		Group B		SMD	95% CI		z	p	Specific weight (%)
		N	Mean±SD	N	Mean±SD		Min	Max			
Early (Group A) and On Time (Group B)	Arede et al. (2018)	15	21.54±10.57	10	20.58±11.86	0.08	-0.72	0.88	0.20	0.837	14.8
	López-Plaza et al. (2016) - 1	44	9.19±6.43	36	7.84±6.34	0.21	-0.23	0.65	0.93	0.353	48.6
	López-Plaza et al. (2016) - 2	30	5.69±8.24	30	3.17±7.84	0.31	-0.20	0.82	1.19	0.233	36.6
	Pooled standardized mean differences:					0.23	-0.08	0.54	1.45	0.148	100
	Authors	Group A		Group C		SMD	95% CI		z	p	Specific weight (%)
		N	Mean±SD	N	Mean±SD		Min	Max			
Early (Group A) and Late (Group C)	Arede et al. (2018)	15	21.54±10.57	9	24.9±8.85	-0.34	-1.17	0.50	0.79	0.427	22.1
	López-Plaza et al. (2016) - 1	44	9.19±6.43	9	7.58±5.52	0.26	-0.46	0.97	0.70	0.485	29.7
	López-Plaza et al. (2016) - 2	30	5.69±8.24	22	0.52±7.75	0.64	0.08	1.21	2.23	0.025	48.2
	Pooled standardized mean differences:					0.31	-0.08	0.70	1.56	0.11947	100
	Authors	Group B		Group C		SMD	95% CI		z	p	Specific weight (%)
		N	Mean±SD	N	Mean±SD		Min	Max			
On time (Group B) and Late (Group C)	Arede et al. (2018)	10	20.58±11.86	9	24.9±8.85	-0.41	-1.32	0.50	0.88	0.378	16.1
	Gouvea et al. (2016)	18	28.00±6.60	6	31.40±7.40	-0.50	-1.44	0.43	1.05	0.294	15.3
	López-Plaza et al. (2016) - 1	36	7.84±6.34	9	7.58±5.52	0.04	-0.69	0.77	0.11	0.910	25.1
	López-Plaza et al. (2016) - 2	30	3.17±7.84	22	0.52±7.75	0.34	-0.21	0.89	1.20	0.229	43.5
	Pooled standardized mean differences:					0.02	-0.35	0.38	0.09	0.931	100

López-Plaza et al. (2016) – 1: kayakers; López-Plaza et al. (2016) – 2: canoeists.

Kinanthropometric variables: Male

Table S9. Comparison between maturational groups for height (cm).

	Authors	Group A		Group B		SMD	95% CI		z	p	Specific weight (%)
		N	Mean±SD	N	Mean±SD		Min	Max			
Early (Group A) and On Time (Group B)	Arede et al. (2018)	15	187.35±5.11	10	179.45±6.05	1.39	0.49	2.29	3.01	0.002	6.6
	Carling et al. (2012)	34	171.00±6.20	98	163.50±8.20	0.96	0.56	1.37	4.63	<0.001	9.1
	Figueiredo et al. (2009) - 1	25	148.40±7.30	45	144.60±5.90	0.58	0.09	1.08	2.30	0.021	8.7
	Figueiredo et al. (2009) - 2	23	169.10±6.00	45	162.20±8.80	0.86	0.33	1.38	3.20	0.001	8.5
	Guimaraes et al. (2019)	30	177.90±5.70	84	164.30±7.80	1.85	1.36	2.33	7.49	<0.001	8.7
	López-Plaza et al. (2016) - 1	44	172.94±4.75	36	165.70±4.65	1.52	1.02	2.03	5.94	<0.001	8.6
	López-Plaza et al. (2016) - 2	30	170.73±5.56	30	162.30±7.17	1.30	0.74	1.86	4.54	<0.001	8.4
	Matta et al. (2014)	41	167.90±6.80	41	165.20±7.30	0.38	-0.06	0.82	1.70	0.089	9.0
	Matthys et al. (2012)	13	182.60±7.60	135	171.60±5.90	1.81	1.20	2.41	5.84	<0.001	8.1
	Valente-Dos Santos et al. (2014) - 2	8	155.30±7.00	37	151.60±6.80	0.53	-0.24	1.30	1.35	0.177	7.3
	Valente-Dos Santos et al. (2014) - 3	21	160.10±7.60	62	161.40±6.40	-0.19	-0.69	0.30	0.76	0.448	8.7
	Valente-Dos Santos et al. (2014) - 4	21	167.10±6.10	27	168.00±6.10	-0.15	-0.72	0.43	0.50	0.618	8.3
	Pooled standardized mean differences:					0.90	0.50	1.29	4.44	<0.001	100

	Authors	Group A		Group C		SMD	95% CI		z	p	Specific weight (%)
		N	Mean±SD	N	Mean±SD		Min	Max			
Early (Group A) and Late (Group C)	Arede et al. (2018)	15	187.35±5.11	9	170.70±3.76	3.45	2.10	4.80	5.01	<0.001	7.1
	Carling et al. (2012)	34	171.00±6.20	26	152.90±5.10	3.11	2.34	3.87	7.92	<0.001	8.5
	Figueiredo et al. (2009) - 1	25	148.40±7.30	17	139.40±4.50	1.39	0.70	2.09	3.96	<0.001	8.7
	Gastin et al. (2013)	25	168.50±11.30	25	159.50±11.80	0.77	0.19	1.34	2.61	0.009	8.9
	Guimaraes et al. (2019)	30	177.90±5.70	36	154.50±7.40	3.46	2.68	4.24	8.72	<0.001	8.5
	Hammami et al. (2017)	22	175.05±5.12	34	156.71±7.00	2.85	2.09	3.62	7.29	<0.001	8.5
	López-Plaza et al. (2016) - 1	44	172.94±4.75	9	158.30±5.21	2.99	2.06	3.92	6.30	<0.001	8.2
	López-Plaza et al. (2016) - 2	30	170.73±5.56	22	153.13±7.69	2.65	1.89	3.41	6.80	<0.001	8.5
	Matta et al. (2014)	41	167.90±6.80	32	159.10±9.90	1.05	0.56	1.54	4.16	<0.001	9.0
	Matthys et al. (2012)	13	182.60±7.60	20	157.80±5.90	3.66	2.49	4.83	6.12	<0.001	7.6
	Valente-Dos Santos et al. (2014) - 2	8	155.30±7.00	8	149.10±38.50	0.21	-0.77	1.20	0.42	0.672	8.0
	Valente-Dos Santos et al. (2014) - 3	21	160.10±7.60	8	161.50±52.70	-0.05	-0.86	0.77	0.12	0.905	8.4
	Pooled standardized mean differences:					2.09	1.37	2.81	5.67	<0.001	100

	Authors	Group B		Group C		SMD	95% CI		z	p	Specific weight (%)
		N	Mean±SD	N	Mean±SD		Min	Max			
On time (Group B) and Late [illegible]	Arede et al. (2018)	10	179.45±6.05	9	170.70±3.76	1.64	0.56	2.71	2.99	0.002	5.9
	Carling et al. (2012)	98	163.50±8.20	26	152.90±5.10	1.37	0.91	1.84	5.78	<0.001	9.9
	Figueiredo et al. (2009) - 1	45	144.60±5.90	17	139.40±4.50	0.92	0.34	1.51	3.11	0.001	9.1

Gouvea et al. (2016)	18	160.70±11.00	6	142.00±4.10	1.83	0.75	2.91	3.31	<0.001	5.8
Guimaraes et al. (2019)	84	164.30±7.80	36	154.50±7.40	1.27	0.84	1.69	5.87	<0.001	10.2
López-Plaza et al. (2016) - 1	36	165.70±4.65	9	158.30±5.21	1.53	0.73	2.33	3.73	<0.001	7.5
López-Plaza et al. (2016) - 2	30	162.30±7.17	22	153.13±7.69	1.22	0.62	1.82	3.98	<0.001	8.9
Matta et al. (2014)	41	165.20±7.30	32	159.10±9.90	0.71	0.23	1.18	2.91	0.003	9.8
Matthys et al. (2012)	135	171.60±5.90	20	157.80±5.90	2.33	1.79	2.87	8.48	<0.001	9.4
Valente-Dos Santos et al. (2014) - 1	22	146.90±6.20	10	117.50±70.60	0.73	-0.04	1.51	1.87	0.062	7.7
Valente-Dos Santos et al. (2014) - 2	37	151.60±6.80	8	149.10±38.50	0.15	-0.62	0.91	0.38	0.706	7.8
Valente-Dos Santos et al. (2014) - 3	62	161.40±6.40	8	161.50±52.70	-0.01	-0.74	0.73	0.01	0.988	8.0
Pooled standardized mean differences:					1.13	0.76	1.50	6.04	<0.001	100

Figueiredo et al. (2009) – 1: 11.0 to 12.9 years-old; Figueiredo et al. (2009) – 2: 13.0 to 14.9 years-old; López-Plaza et al. (2016) – 1: kayakers; López-Plaza et al. (2016) – 2: canoeists; Valente-Dos Santos et al. (2014) – 1: 12 years-old; Valente-Dos Santos et al. (2014) – 2: 13 years-old; Valente-Dos Santos et al. (2014) – 3: 14 years-old; Valente-Dos Santos et al. (2014) – 4: 15 years-old.

Table S10. Comparison between maturational groups for body mass (kg).

	Authors	Group A		Group B		SMD	95% CI		z	p	Specific weight (%)
		N	Mean±SD	N	Mean±SD		Min	Max			
Early (Group A) and On Time (Group B)	Arede et al. (2018)	15	78.63±7.79	10	69.13±7.20	1.21	0.33	2.09	2.70	0.006	7.0
	Carling et al. (2012)	34	60.60±6.60	98	52.00±8.20	1.09	0.68	1.51	5.19	<0.001	12.8
	Figueiredo et al. (2009) - 1	25	42.10±7.10	45	37.50±5.10	0.77	0.27	1.28	2.99	0.002	11.5
	Figueiredo et al. (2009) - 2	23	60.20±8.70	45	52.30±9.00	0.88	0.35	1.40	3.28	0.001	11.2
	Guimaraes et al. (2019)	30	65.70±7.60	84	54.80±9.00	1.25	0.80	1.70	5.47	<0.001	12.3
	López-Plaza et al. (2016) - 1	44	64.78±8.66	36	56.35±6.80	1.06	0.59	1.53	4.40	<0.001	12.0
	López-Plaza et al. (2016) - 2	30	64.82±8.60	30	54.36±9.63	1.13	0.58	1.68	4.05	<0.001	10.9
	Matta et al. (2014)	41	58.20±12.30	41	54.50±9.10	0.34	-0.10	0.77	1.52	0.127	12.5
	Matthys et al. (2012)	13	76.20±8.90	135	58.40±8.00	2.19	1.57	2.82	6.90	<0.001	9.9
	Pooled standardized mean differences:					1.07	0.77	1.38	6.84	<0.001	100

	Authors	Group A		Group C		SMD	95% CI		z	p	Specific weight (%)
		N	Mean±SD	N	Mean±SD		Min	Max			
Early (Group A) and Late (Group C)	Arede et al. (2018)	15	78.63±7.79	9	57.58±6.34	2.79	1.59	3.98	4.57	<0.001	8.9
	Carling et al. (2012)	34	60.60±6.60	26	40.60±3.40	3.62	2.78	4.46	8.42	<0.001	10.0
	Figueiredo et al. (2009) - 1	25	42.10±7.10	17	33.60±3.60	1.40	0.71	2.09	3.97	<0.001	10.4
	Gastin et al. (2013)	25	58.20±13.60	25	53.40±15.40	0.33	-0.23	0.88	1.14	0.253	10.7
	Guimaraes et al. (2019)	30	65.70±7.60	36	43.10±7.30	3.00	2.29	3.72	8.21	<0.001	10.3
	Hammami et al. (2017)	22	66.70±8.92	34	48.33±9.77	1.92	1.27	2.57	5.78	<0.001	10.5
	López-Plaza et al. (2016) - 1	44	64.78±8.66	9	48.40±6.27	1.94	1.12	2.75	4.67	<0.001	10.1
	López-Plaza et al. (2016) - 2	30	64.82±8.60	22	42.97±6.55	2.76	1.98	3.54	6.94	<0.001	10.1
	Matta et al. (2014)	41	58.20±12.30	32	48.60±8.80	0.87	0.39	1.36	3.52	<0.001	10.9
	Matthys et al. (2012)	13	76.20±8.90	20	43.00±5.20	4.71	3.31	6.11	6.61	<0.001	8.2
	Pooled standardized mean differences:					2.26	1.49	3.02	5.81	<0.001	100

	Authors	Group B		Group C		SMD	95% CI		z	p	Specific weight (%)
		N	Mean±SD	N	Mean±SD		Min	Max			
On time (Group B) and Late (Group C)	Arede et al. (2018)	10	69.13±7.20	9	57.58±6.34	1.62	0.55	2.69	2.97	0.003	5.8
	Carling et al. (2012)	98	52.00±8.20	26	40.60±3.40	1.52	1.04	1.99	6.28	<0.001	14.0
	Figueiredo et al. (2009) - 1	45	37.50±5.10	17	33.60±3.60	0.81	0.23	1.39	2.76	0.005	12.0
	Gouvea et al. (2016)	18	49.30±11.90	6	34.50±3.00	1.35	0.34	2.37	2.61	0.008	6.2
	Guimaraes et al. (2019)	84	54.80±9.00	36	43.10±7.30	1.36	0.93	1.79	6.24	<0.001	14.9
	López-Plaza et al. (2016) - 1	36	56.35±6.80	9	48.40±6.27	1.16	0.39	1.94	2.96	0.003	8.9
	López-Plaza et al. (2016) - 2	30	54.36±9.63	22	42.97±6.55	1.32	0.71	1.93	4.25	<0.001	11.4
	Matta et al. (2014)	41	54.50±9.10	32	48.60±8.80	0.65	0.18	1.13	2.69	0.007	13.9
	Matthys et al. (2012)	135	58.40±8.00	20	43.00±5.20	1.99	1.47	2.51	7.49	<0.001	13.0
	Pooled standardized mean differences:					1.29	0.99	1.59	8.41	<0.001	100

Figueiredo et al. (2009) – 1: 11.0 to 12.9 years-old; Figueiredo et al. (2009) – 2: 13.0 to 14 .9 years-old; López-Plaza et al. (2016) – 1: kayakers; López-Plaza et al. (2016) – 2: canoeists.

Table S11. Comparison between maturational groups fat mass percentage (%).

	Authors	Group A		Group B		SMD	95% CI		z	p	Specific weight (%)
		N	Mean±SD	N	Mean±SD		Min	Max			
Early (Group A) and On Time (Group B)	Carling et al. (2012)	34	12.90±2.50	98	12.40±2.20	0.22	-0.17	0.61	1.09	0.274	30.8
	López-Plaza et al. (2016) - 1	44	16.39±6.39	36	15.47±4.64	0.16	-0.28	0.60	0.71	0.475	27.1
	López-Plaza et al. (2016) - 2	30	18.16±9.00	30	15.43±7.36	0.33	-0.18	0.84	1.26	0.207	22.8
	Matthys et al. (2012)	13	15.60±3.40	135	11.70±4.20	0.94	0.36	1.52	3.17	0.001	19.3
	Pooled standardized mean differences:					0.37	0.06	0.67	2.33	0.019	100
	Authors	Group A		Group C		SMD	95% CI		z	p	Specific weight (%)
		N	Mean±SD	N	Mean±SD		Min	Max			
Early (Group A) and Late (Group C)	Carling et al. (2012)	34	12.90±2.50	26	11.50±2.50	0.55	0.03	1.07	2.08	0.037	21.7
	Hammami et al. (2017)	22	12.26±6.41	34	14.45±6.47	-0.33	-0.88	0.21	1.22	0.224	21.4
	López-Plaza et al. (2016) - 1	44	16.39±6.39	9	15.11±6.05	0.20	-0.52	0.92	0.54	0.587	18.7
	López-Plaza et al. (2016) - 2	30	18.16±9.00	22	12.30±4.45	0.78	0.20	1.35	2.66	0.007	20.9
	Matthys et al. (2012)	13	15.60±3.40	20	9.10±4.40	1.57	0.76	2.38	3.81	<0.001	17.4
	Pooled standardized mean differences:					0.52	-0.06	1.09	1.77	0.076	100
	Authors	Group B		Group C		SMD	95% CI		z	p	Specific weight (%)
		N	Mean±SD	N	Mean±SD		Min	Max			
On time (Group B) and Late (Group C)	Carling et al. (2012)	98	12.40±2.20	26	11.50±2.50	0.39	-0.04	0.83	1.78	0.075	33.2
	Gouvea et al. (2016)	18	11.70±3.20	6	8.70±4.00	0.85	-0.11	1.81	1.74	0.082	6.8
	López-Plaza et al. (2016) - 1	36	15.47±4.64	9	15.11±6.05	0.07	-0.66	0.80	0.19	0.847	11.8
	López-Plaza et al. (2016) - 2	30	15.43±7.36	22	12.30±4.45	0.49	-0.07	1.05	1.72	0.086	20.2
	Matthys et al. (2012)	135	11.70±4.20	20	9.10±4.40	0.61	0.14	1.09	2.53	0.011	28.0
	Pooled standardized mean differences:					0.47	0.22	0.72	3.65	<0.001	100

López-Plaza et al. (2016) – 1: kayakers; López-Plaza et al. (2016) – 2: canoeists.

Table S12. Comparison between maturational groups for sitting heigh (cm)t.

	Authors	Group A		Group B		SMD	95% CI		z	p	Specific weight (%)
		N	Mean±SD	N	Mean±SD		Min	Max			
Early (Group A) and On Time (Group B)	Figueiredo et al. (2009) - 1	25	75.00±2.70	45	72.70±2.70	0.84	0.33	1.35	3.24	0.001	25.8
	Figueiredo et al. (2009) - 2	23	85.50±3.60	45	80.30±4.20	1.28	0.73	1.83	4.57	<0.001	25.3
	López-Plaza et al. (2016) - 1	44	92.91±2.64	36	86.89±2.19	2.44	1.85	3.02	8.14	<0.001	24.8
	López-Plaza et al. (2016) - 2	30	91.17±2.90	30	85.77±2.30	2.04	1.41	2.67	6.33	<0.001	24.1
	Pooled standardized mean differences:					1.64	0.92	2.36	4.46	<0.001	100
	Authors	Group A		Group C		SMD	95% CI		z	p	Specific weight (%)
		N	Mean±SD	N	Mean±SD		Min	Max			
Early (Group A) and Late (Group C)	Figueiredo et al. (2009) - 1	25	75.00±2.70	17	70.60±2.50	1.65	0.93	2.37	4.49	<0.001	25.7
	Gastin et al. (2013)	25	92.00±3.60	25	87.40±4.20	1.16	0.55	1.76	3.76	<0.001	26.2
	López-Plaza et al. (2016) - 1	44	92.91±2.64	9	82.07±2.83	4.00	2.93	5.07	7.34	<0.001	23.6
	López-Plaza et al. (2016) - 2	30	91.17±2.90	22	79.60±3.42	3.64	2.73	4.55	7.82	<0.001	24.6
	Pooled standardized mean differences:					2.56	1.22	3.91	3.74	<0.001	100
	Authors	Group B		Group C		SMD	95% CI		z	p	Specific weight (%)
		N	Mean±SD	N	Mean±SD		Min	Max			
On time (Group B) and Late (Group C)	Figueiredo et al. (2009) - 1	45	72.70±2.70	17	70.60±2.50	0.78	0.21	1.36	2.67	0.007	35.7
	López-Plaza et al. (2016) - 1	36	86.89±2.19	9	82.07±2.83	2.04	1.19	2.89	4.68	<0.001	30.8
	López-Plaza et al. (2016) - 2	30	85.77±2.30	22	79.60±3.42	2.15	1.45	2.85	6.04	<0.001	33.6
	Pooled standardized mean differences:					1.63	0.69	2.57	3.39	<0.001	100

Figueiredo et al. (2009) – 1: 11.0 to 12.9 years-old; Figueiredo et al. (2009) – 2: 13.0 to 14 .9 years-old; López-Plaza et al. (2016) – 1: kayakers; López-Plaza et al. (2016) – 2: canoeists.

Table S13. Comparison between maturational groups for BMI (kg/m²).

Early (Group A) and On Time (Group [illegible]	Authors	Group A N	Group A Mean±SD	Group B N	Group B Mean±SD	SMD	95% CI Min	95% CI Max	z	p	Specific weight (%)
	López-Plaza et al. (2016) - 1	44	21.63±2.49	36	20.49±1.98	0.50	0.05	0.94	2.17	0.029	57.3
	López-Plaza et al. (2016) - 2	30	22.22±2.69	30	20.55±2.79	0.60	0.08	1.12	2.28	0.022	42.7
	Pooled standardized mean differences:					0.54	0.20	0.88	3.13	0.001	100

Early (Group A) and Late (Group C)	Authors	Group A N	Group A Mean±SD	Group C N	Group C Mean±SD	SMD	95% CI Min	95% CI Max	z	p	Specific weight (%)
	López-Plaza et al. (2016) - 1	44	21.63±2.49	9	19.27±1.79	0.97	0.23	1.71	2.56	0.010	46.6
	López-Plaza et al. (2016) - 2	30	22.22±2.69	22	18.25±1.68	1.69	1.04	2.33	5.12	<0.001	53.4
	Pooled standardized mean differences:					1.35	0.65	2.05	3.80	<0.001	100

On time (Group B) and Late (Group C)	Authors	Group B N	Group B Mean±SD	Group C N	Group C Mean±SD	SMD	95% CI Min	95% CI Max	z	p	Specific weight (%)
	López-Plaza et al. (2016) - 1	36	20.49±1.98	9	19.27±1.79	0.62	-0.13	1.36	1.63	0.103	38.1
	López-Plaza et al. (2016) - 2	30	20.55±2.79	22	18.25±1.68	0.95	0.37	1.53	3.20	0.001	61.9
	Pooled standardized mean differences:					0.82	0.36	1.28	3.52	<0.001	100

BMI: Body mass index; López-Plaza et al. (2016) – 1: kayakers; López-Plaza et al. (2016) – 2: canoeists.

Kinanthropometric variables: Female.

Table S14. Comparison between early and late maturers groups for height (cm).

	Authors	Group A		Group C		SMD	95% CI		z	p	Specific weight (%)
		N	Mean±SD	N	Mean±SD		Min	Max			
Early (Group A) and Late (Group C	Leonardi et al. (2018)	27	166.00±6.22	12	158.10±9.79	1.04	0.32	1.76	2.82	0.004	37.1
	Sogut et al. (2019)	30	157.50±6.80	31	148.80±6.60	1.28	0.73	1.84	4.53	<0.001	62.9
Pooled standardized mean differences:						1.19	0.75	1.63	5.31	<0.001	100

Table S15. Comparison between early and late maturers groups for body mass (kg).

	Authors	Group A		Group C		SMD	95% CI		z	p	Specific weight (%)
		N	Mean±SD	N	Mean±SD		Min	Max			
Early (Group A) and Late (Group C)	Leonardi et al. (2018)	27	61.80±9.79	12	48.90±11.20	1.24	0.50	1.97	3.28	0.001	33.5
	Sogut et al. (2019)	30	47.80±10.40	31	40.60±6.40	0.83	0.30	1.35	3.09	0.002	66.5
Pooled standardized mean differences:						0.96	0.54	1.39	4.42	<0.001	100

Table S16. Comparison between early and late maturers groups for BMI (kg/m^2).

	Authors	Group A		Group C		SMD	95% CI		z	p	Specific weight (%)
		N	Mean±SD	N	Mean±SD		Min	Max			
Early (Group A) and Late (Group C)	Leonardi et al. (2018)	27	22.20±3.04	12	19.10±3.08	1.00	0.28	1.71	2.71	0.006	44.5
	Sogut et al. (2019)	30	18.80±3.50	31	18.20±2.00	0.21	-0.29	0.71	0.81	0.416	55.5
Pooled standardized mean differences:						0.56	-0.21	1.32	1.43	0.153	100

BMI: Body mass index.
